# Comment: Social Psychiatry: Is it going out of fashion?

**DOI:** 10.1177/00207640221116301

**Published:** 2022-08-17

**Authors:** Sam Nishanth Gnanapragasam

**Affiliations:** South London and Maudsley NHS Foundation Trust, UK

The International Journal of Social Psychiatry has sought to provide a forum to share findings related to social psychiatry since 1954 since it was established by Joshua Bierer. This has been done with an explicit and consensus understanding that the impact of social factors is profound on the wellbeing and mental health of individuals around the world.

## The case for social psychiatry

Humans are fundamentally social being. Their relationships and how they interact with the world can cause difficulties as well as protect from difficulties. Social factors are therefore key determinants for physical and mental health wellbeing (Marmot & Wilkinson, 2005). Mental health disorders that result are ‘intrinsically social phenomenon’ (Bhugra & Morgan, 2010), both in terms of social context shape them and how they then resultantly shape the social context.

Social psychiatry is a field of psychiatry that considers the influences of culture, social and environmental factors in the aetiology as well outcomes and treatment for mental health conditions impacting individuals. It considers and seeks to link psychiatry with the important disciplines of sociology, anthropology and others, and is importantly influenced by them. The case for this field is made by Ventriglio et al. (2016) who forcefully answer the question ‘Why do we need a social psychiatry’.

## Cause for concern

In this context, Dr. Alibudbud’s paper ‘Decreasing public interest in social psychiatry: An infodemiological study of worldwide Google search volumes from 2004 to 2021’ (Alibudbud, 2022) in this issue. It has prompted some important considerations – is social psychiatry still relevant in today’s age? Could it be that the focus of this journal, social psychiatry, is going out of fashion? Should we use a different term that is more fit for the modern age?

Alibudbud’s research outlines reason for concern – namely the decline in volume of searches for social psychiatry between 2004 and 2021. Whilst this may be a somewhat crude measure, this does echo concerns raised by others around the decline of social psychiatry and that it has perhaps seen ‘better days’ (Pilgrim & Rogers, 2005). At the same time, it could be that the concepts underpinning social psychiatry are recognised and increasingly used in discourse, however that different terms are being used instead – namely, public mental health, cultural psychiatry, global mental health and social determinants of mental health.

## What should happen?

We posit that social psychiatry is more important than ever to help us truly understand, support and improve the mental health of individuals around the world. This has become clearer through the course of the Covid-19 pandemic with differential physical and mental health outcomes owing to gender, ethnicity and socio-economic status. Often, an intersection between these factors. In addition, there is a strong case for modernisation and strengthening the field of social psychiatry – for example, with considering geopolitical influencers on health in the emerging area of geopsychiatry (Castaldelli-Maia & Bhugra, 2022).

It is worth supporting the recommendation put forward by Dr. Alibudbud that there is a need to improve mental health and psychiatry’s connection to social sciences, and renew the offer of social psychiatry for this moment in the 21st century. Social psychiatry is an important part of the future of psychiatry and the growing area of public mental health. Humans are social, and therefore psychiatry must be so too. It is important to embed the underpinning concepts of social psychiatry in undergraduate and post-graduate training so that the profession can appropriately advocate for those most vulnerable and ensure delivery of social interventions to improve the social context and determinants of mental health.
